# Association between Family and Friend Smoking Status and Adolescent Smoking Behavior and E-Cigarette Use in Korea

**DOI:** 10.3390/ijerph13121183

**Published:** 2016-11-25

**Authors:** Myoung Jin Joung, Mi Ah Han, Jong Park, So Yeon Ryu

**Affiliations:** 1 Department of Public Health, Graduate School of Chosun University, 309 Pilmun-daero, Dong-gu, Gwangju 61452, Korea; danjack@daum.net; 2 Department of Preventive Medicine, College of Medicine, Chosun University, 309 Pilmun-daero, Dong-gu, Gwangju 61452, Korea; jpark@chosun.ac.kr (J.P.); canrsy@chosun.ac.kr (S.Y.R.)

**Keywords:** adolescent, electronic cigarettes, family, friends, smoking, tobacco

## Abstract

Smoking is harmful to the health of adolescents because their bodies are still growing. The aim of this study was to analyze the association between the smoking status of Korean adolescents’ parents and friends and their own smoking behavior. The study assessed a nationwide sample of 72,060 middle and high students from the 10th Korea Youth Risk Behavior Web-based Survey (2014). Descriptive analysis, chi-square tests, and multiple logistic regression analysis were used to probe the association between family and friend smoking status and adolescent smoking behavior. The current cigarette smoking rates were 13.3% of boys and 4.1% of girls. The corresponding rates for electronic cigarette smoking were 4.1% and 1.5%, respectively. Higher exposure to secondhand smoke, smoking by any family member, more friends smoking, and witnessed smoking at school were associated with current smoking and electronic smoking. The smoking status of family and friends was significantly related to adolescent smoking behavior. These results should be considered in designing programs to control adolescent smoking.

## 1. Introduction

Globally, smoking increases rates of disease and premature death [1]. It has been reported that 22% of 8th graders and 46% of 12th graders tried smoking despite considerable U.S. public health efforts to prevent adolescent smoking [2]. In Korea, in 2013, the prevalence of cigarette smoking was greater among boys (14.4%) than girls (4.6%) in middle and high school, and prevalence of current cigarette smoking among adolescent males was greater than the Health Plan 2020 target of 12.0% [3].

Smoking can be especially damaging to the health of adolescents because their bodies are still developing; exposure to nicotine, tar, carbon monoxide, and other carcinogens triggers the development of chronic diseases and causes greater cell and tissue damage than they would in adults [4]. In addition, adolescent smokers are more likely to keep smoking throughout their lifetime. Early age smoking may lead to other delinquencies such as substance use, dropping out of school, sexual risk taking, and violence [5].

Adolescents are susceptible to influence by social and environmental factors, and family history, companionship, personal characteristics, and psycho-social and psycho-pathological problems may motivate adolescents to smoke [6]. Peer and familial influences on adolescent smoking behavior have already been demonstrated [7,8]. There is a greater chance that children living with smoking parents will smoke and a smaller possibility that they will quit [9]. When there is coercive pressure to perform risky behaviors, peers have a profound effect on each other and may encourage experimentation. With regard to adolescent use of tobacco and alcohol, there is convincing evidence that it is affirmatively associated with their friends’ use or lack of use [8].

In Korea, many previous studies have investigated adolescents’ smoking behavior and the effect of family and friends who smoke. More exposure to secondhand smoking at home was associated with the higher risks of daily smoking, current smoking, and ever smoking in Korea [10]. When adolescents had been exposed to secondhand smoking, the risk of smoking was significantly increased [11]. Peer cigarette smoking had a significant association with e-cigarette use in adolescent nonsmokers [12]. However, the influence of each family members’ smoking on adolescent smoking behavior was limited in Korea.

Recently, the rate of electronic cigarette use has increased in Korean adolescents [13,14]. However, most previous studies of adolescents smoking behavior were focused on tobacco cigarettes [10,11]. Here, we analyzed the relationship between secondhand smoking exposure and the smoking status of friends and their current smoking behavior. We investigated adolescent smoking behavior with respect to smoking status of each family member. The current status of electronic smoking, which has increased recently, and its association with family and friends’ smoking were also investigated.

## 2. Methods

### 2.1. Data Source

This study used data from the 10th Korea Youth Risk Behavior Web-based Survey (KYRBS), 2014, a survey conducted by the Korean Centers for Disease Control and Prevention (KCDC) every year since 2005. In 2014, a three-stage cluster-sample design was used to obtain a nationally representative sample. In the first stage (stratification), the study population was stratified by geographic region and school type. In the second stage (sample allocation), totally, 400 middle schools and 400 high schools were selected by proportional sampling to match the study population. In the third stage (stratified cluster sampling), the sample schools were selected by systematic sampling and sample classes were selected by simple randomization sampling from selected schools [15]. All students in the sampled classes are eligible to participate. The total population of the sample was 74,176.

KYRBS focused on health-risk behaviors including tobacco use, alcohol use, obesity, etc. The 2014 questionnaire consisted of 125 items in 15 domains of health-risk behaviors. The survey was conducted for 45–50 min during a class in a computer room where students can access the Internet. Questionnaire private access keys were allocated to each student and distributed by the teachers. Data were collected using a self-reported method. Participants were not allowed to ask or discuss with teachers or peers and they depended only on their personal understanding of the questionnaire. Ethical approval was obtained from the institutional review board of KCDC (2014-06EXP-02-P-A). A total of 72,060 adolescents participated in the 2014 survey, and the response rate was 97.2% (72,060/74,167). Detailed information of data source is available elsewhere [16].

### 2.2. Variables

#### 2.2.1. General Characteristics

The general characteristics included were school year (middle 1st, middle 2nd, middle 3rd, high 1st, high 2nd, and high 3rd), perceived academic record (high, medium, and low), and perceived economic status (high, medium, and low). Academic record and economic status were collected by subjective assessment. The survey also queried frequency of alcohol consumption within the last 30 days (none, 1–5 days, 6–9 days, and ≥10 days), frequency of intense physical activity during the past 7 days (none, 1–2 days, and ≥3 days), disease history (asthma, allergic rhinitis, and atopic dermatitis), and perceived stress level (high and low). Stress level was measured as following: “How much do you feel stress in your usual life?” The answers were “very much, much, a little bit, not so much, not at all”. Then, the answers were reclassified into 2 groups: high (very much and much) and low (a little bit, not so much, and not at all).

#### 2.2.2. Family and Friends’ Smoking Status

The smoking status of family and friends were secondhand smoke exposure in household (none, 1–2 days, and ≥3 days), family smoking status, friends’ smoking status (none, some, and most/all), and witnessed smoking at school (no and yes). When there were any family members who smoked, they were subcategorized as fathers, mothers, siblings, grandparents, or others.

#### 2.2.3. Current Smoking and Electronic Smoking Status

Adolescent smoking behavior was assessed as current smoking and current electronic smoking. We defined the students who smoked at least 1 day during the past 30 days before the survey as current smokers. Current electronic cigarette use was defined as device use within the past 30 days.

### 2.3. Data Analysis

All data analyses were performed using SPSS software (version 18, SPSS Inc., Chicago, IL, USA). General characteristics of adolescents and smoking status of family and friends by sex were compared with chi-square tests and Cramer’s V (small, medium, and large effect size are 0.10, 0.30, and 0.50, respectively) were calculated. The proportions of current smoking by smoking status of family and friends were calculated by chi-square tests. Finally, multiple logistic regression analysis was used to calculate the odds ratios (ORs) and 95% confidence intervals (95% CIs) for adolescents’ current smoking by smoking status of family and friends after adjusting grade, perceived academic records, perceived socioeconomic status, alcohol drinking frequency, frequency of intense physical activity, disease history, and stress level which considered the associated factors of adolescents smoking behavior. Multicollinearity was checked using the tolerance values and variance inflation factor (VIF). All VIF values were less than 10, which meant there was no multicollinearity. Previous literature reported that there were substantial differences in the smoking behavior and its related factors between boys and girls [12] and stratified analysis by sex were performed. Differences were considered statistically significant at *p* < 0.05.

## 3. Results

### 3.1. General Characteristics by Sex and Smoking Status of Family and Friends by Sex

The proportions of students exposed to secondhand smoke in the household ≥3 days per week were 16.7% of boys and of 19.5% girls, respectively (*p* < 0.001). More than half of subjects had family members who smoked. Among family members, the highest smoking rate was noted for fathers (46.4% of boys and 48.4% of girls). About 15% of boys responded that most/all friends smoked, compared to just 5.0% of girls (*p* < 0.001). The proportions of boy and girl students who reported having witnessed smoking at school were 43.9% and 35.9%, respectively (*p* < 0.001), and effect sizes were small for all comparisons (Table 1).

### 3.2. Adolescent Smoking Status by Family and Friends’ Smoking Status

The current smoking rates were 13.3% of boys and 4.1% of girls. It was significantly higher in those with higher exposure to secondhand smoke. In the case of boys, when the siblings smoked, the current smoking rate was the highest (34.8%) compared to when the father smoked (14.6%) or the mother smoked (29.1%). In the case of girls, when the mother smoked, the current smoking rate was the highest (14.7%). When students reported that most/all of their friends smoked, they were more likely to have current smoking experience. Regarding subjects who witnessed smoking at school, the percentages of boys and girls who had current smoking experience were 19.6% and 6.0%, respectively. The current electronic smoking rates were 7.5% of boys and 1.5% of girls, respectively. With similar current smoking status, higher exposure to secondhand smoke, smoking of any family member, more friends smoking, and witnessed smoking at school were associated with current electronic smoking status in both sexes. All comparisons between family and friends’ smoking and adolescents smoking were statistically significant in both sexes (*p* < 0.05) (Table 2).

### 3.3. Associations for Current Smoking and Current Electronic Smoking with Family and Friends’ Smoking Status

Adolescents exposed to secondhand smoke at home ≥3 days/week had higher ORs for current smoking than adolescents without secondhand smoke exposure (boys: OR = 1.90, 95% CI = 1.75–2.07, girls: OR = 2.06, 95% CI = 1.80–2.35). With respect to any family members who smoked, the ORs of current smoking were significantly higher compared to adolescents without family members who smoked. According to each family member’s smoking status, ORs for current smoking were highest among boys in the sibling group (OR = 2.34, 95% CI = 2.09–2.62) and girls in the mother group (OR = 2.62, 95% CI = 2.15–3.21). Adolescents who reported that “most/all” friends smoked had higher ORs for current smoking than peers who did not smoke. Adolescents of both sexes who witnessed smoking at school had a higher likelihood of current smoking (Table 3).

Adolescents with higher exposure to secondhand smoke in the home had a higher likelihood of current electronic smoking (boys: OR = 1.96, 95% CI = 1.78–2.17, girls: OR = 2.86, 95% CI = 2.34–3.49). For any family members who smoke, the OR of electronic cigarette smoking was the greatest for students in the sibling group (boys: OR = 2.07, 95% CI = 1.83–2.35, girls: OR = 2.49, 95% CI = 2.01–3.10). Adolescents who had “most/all” friends that smoke had higher ORs for current electronic cigarette smoking than their peers who did not smoke (Table 4).

## 4. Discussion

Adolescent smoking is a significant public health concern. We used data from the 10th KYRBS of 2014 to analyze the relationship between the smoking status of Korean adolescents’ parents and friends and their own smoking behavior. In this representative study, 11.2% of boy students and 3.4% of girl students were current smokers. When compared to Organisation for Economic Co-operation and Development (OECD) data of the Swedish population, just 10.7% of people over 15 years were daily smokers, even though the Korean smoking rate was lower than that of the average OECD countries (16.0%) [17]. Notably, the proportion of boys who were current smokers was higher than that of the U.K. (10.0%) and Canada (8%).

In the present study, the OR of adolescents’ current smoking was higher in adolescents with any family members who smoked compared to adolescents without smoking family members. Parenting continues to be an important factor related to adolescent smoking; other studies have also reported that if parents smoke, their children are more likely to smoke [7,18]. In 20 school districts in Washington State, 31.8% and 18.6% of 12th graders smoked if a parent did or did not, respectively [19]. The ORs for current smoking were substantially different according to each family member’s smoking status. The ORs of adolescents’ current smoking experience were highest among boys when their sibling smoked (OR = 2.34, 95% CI = 2.09–2.62) and girls when their mother smoked (OR = 2.62, 95% CI = 2.15–3.21), respectively. Previous meta-analysis provided the magnitude of the effects of smoking by different family members. The influential effect of smoking on the adolescent that had parents who smoked was stronger with the mother than the father [20]. If no parent smoked and an older sibling smoked, the OR that the 12th grader would smoke was 1.85 compared to 1.49 if a parent smoked [21]. Intentions to smoke and smoking-related attitudes were influenced by family and friend smoking status [22] and family structure affected the adolescents smoking stages [23]. These findings demonstrate that family smoking is a crucial factor that affects adolescent smoking behavior. When it comes to family smoking, relationships between adolescents and family members need to be considered.

The current study shows that if adolescents have friends who smoke, they are more likely to smoke. Others have found that peer smoking was related to both adolescent smoking and initiation of smoking [9]. The present study did not consider the interactions and relationships between the students and their friends because a friendship variable was not included in the survey. One group suggested that mutual or reciprocated types of friend relationships have stronger effects on adolescent smoking behaviors than directional relationships [24]. Further research needs to be done to assess the influence of other relationship types such as friendships on adolescent smoking behavior.

Our study revealed that adolescents were more likely to smoke if they had witnessed smoking at school. This population can be directly and indirectly influenced by their school environment. Direct approval happens when students smoked themselves, sending the message that it is acceptable to smoke. Indirect approval occurs when a person had witnessed smoking behavior in others around them and accepts it (i.e., teachers smoking in the staff room or on school grounds where students could smell it or be aware of it happening) [25]. Adolescents are easily affected by school environments because they spend a lot of time there. Previous research has demonstrated that smoke-free school environments have a lower prevalence of smoking and less overall cigarette consumption than schools with minimal guidelines [26]. Collectively, the evidence suggests that smoke-free school environments are important to control adolescents’ smoking behaviors.

The associations between smoking exposure and adolescents’ smoking behaviors showed some difference according to cigarette smoking or electronic cigarette smoking. Previous study elucidated that there were different contexts to use and cessation of cigarette smoking and electronic cigarette smoking. Smokers trying to switch over to e-cigarette use may be easily influenced into smoking cigarettes by friends and family members who smoke cigarettes [27]. Although we cannot explain clearly the difference in cigarette and electronic cigarette use in this study, we thought there were different contexts of electronic cigarette use compared to cigarette smoking and future study is needed to determine this point.

There were several limitations of this study. Firstly, the results could be biased due to the self-reported survey format. For example, some questions might have been answered in a socially acceptable manner, especially smoking, so some students may have reported lower or higher frequencies. Non-response can also lead to bias. The participation rate of KYRBS was quite high (97.2%) and question specific non-response rate was within 2%. We thought that the higher the response rate of this survey, the lower the risk of non-response bias. Secondly, since this research was based on a cross-sectional study, it was not possible to examine a possible cause-and-effect relationship. Still, it could be that adolescent smokers have a tendency to become close to friends who smoke. Thirdly, we did not assess students’ popularity. According to some studies, smoking is related to adolescents’ popularity among their peer group (i.e., they may have a tendency to copy popular students) [7,28]. In recent review, friendship networks among adolescents promoted their risky behavior including smoking [29]. Further study is recommended to investigate adolescents’ smoking behavior considering this point. Finally, the survey did not include a parenting style variable, but it has been shown that boys without parental control may have a tendency for substance use. For girls, the quality of the relationship between their parents and themselves is more relevant [21].

## 5. Conclusions

This representative study analyzed adolescent smoking behavior in Korea with a focus on middle and high school students nationwide. Furthermore, KYRBS procedure protects respondents’ privacy, which contributed to detection of greater prevalence of delinquent behaviors (e.g., smoking prevalence) in KYRBS than in other interview surveys (e.g., Korea National Health and Nutrition Examination Survey) for Korean adolescents [16]. We considered electronic cigarette use as well as cigarette smoking, which has increased in Korean adolescents. We examined how each individual family member who smoked influenced student smoking behavior as well as secondhand smoking. A smoke-free environment should be provided for adolescents. This means that adolescents should not only avoid secondhand smoking, but also should be protected from smokers.

## Figures and Tables

**Table 1:** General characteristics by sex and smoking status of family and friends.

Characteristic	Boys	Girls	*p*	Cramer’s V
	(*n* = 36,470)	(*n* = 35,590)		
School Year	-	-	<0.001	0.022
Middle 1st	6078 (16.7)	5583 (15.7)	-	-
Middle 2nd	6331 (17.4)	5944 (16.7)	-	-
Middle 3rd	6154 (16.9)	6066 (17.0)	-	-
High 1st	6048 (16.6)	5776 (16.2)	-	-
High 2nd	6009 (16.5)	6143 (17.3)	-	-
High 3rd	5850 (16.0)	6078 (17.1)	-	-
Perceived Academic Record	-	-	<0.001	0.036
High	13,660 (37.5)	13,063 (36.7)	-	-
Medium	18,471 (50.6)	18,995 (53.5)	-	-
Low	4339 (11.9)	3532 (9.9)	-	-
Perceived Economic Status	-	-	<0.001	0.061
High	13,143 (36.0)	10,802 (30.4)	-	-
Middle	16,906 (46.4)	18,134 (51.0)	-	-
Low	6421 (17.6)	6654 (18.7)	-	-
Alcohol Drinking Frequency	-	-	<0.001	0.098
None	29,349 (80.5)	31,099 (87.4)	-	-
1–5 Days	5412 (14.8)	3686 (10.4)	-	-
6–9 Days	818 (2.2)	383 (1.1)	-	-
≥10 Days	891 (2.4)	422 (1.2)	-	-
Frequency of Intense Physical Activity	-	-	<0.001	0.288
None	4978 (13.6)	11,824 (33.2)	-	-
1–2/Days	13,442 (36.9)	14,883 (41.8)	-	-
≥3/Days	18,050 (49.5)	8883 (25.0)	-	-
Disease History	-	-	<0.001	0.044
No	19,607 (53.8)	17,578 (49.4)	-	-
Yes	16,863 (46.2)	18,012 (50.6)	-	-
Stress level	-	-	<0.001	0.137
High	11,124 (30.5)	15,580 (43.8)	-	-
Low	25,346 (69.5)	20,010 (56.2)	-	-
Secondhand Smoke Exposure in Household (Week)	-	-	<0.001	0.037
None	24,374 (66.8)	22,934 (64.4)	-	-
1–2 Days	6013 (16.5)	5713 (16.1)	-	-
≥3 Days	6083 (16.7)	6943 (19.5)	-	-
Family Smoking Status	-	-	<0.001	0.028
No	15,991 (43.8)	14,618 (41.1)	-	-
Yes ^a^	20,479 (56.2)	20,972 (58.9)	-	-
Father	16,934 (46.4)	17,242 (48.4)	-	-
Mother	1061 (2.9)	1297 (3.6)	-	-
Siblings	2169 (5.9)	2348 (6.6)	-	-
Grandparents	2320 (6.4)	2698 (7.6)	-	-
Others	2374 (6.5)	2284 (6.4)	-	-
Friends’ Smoking Status	-	-	<0.001	0.296
None	14,940 (41.0)	24,761 (69.6)	-	-
Some	15,897 (43.6)	9054 (25.4)	-	-
Most/All	5633 (15.4)	1775 (5.0)	-	-
Witnessed Smoking at School	-	-	<0.001	0.082
No	20,455 (56.1)	22,807 (64.1)	-	-
Yes	16,015 (43.9)	12,783 (35.9)	-	-

Data are expressed as number (%), ^a^ Any family members who smoke.

**Table 2:** Proportion of current smoking by family and friends’ smoking status ^a^.

Characteristic	Current Smoking				Current Electronic Smoking			
	Boys	Cramer’s V	Girls	Cramer’s V	Boys	Cramer’s V	Girls	Cramer’s V
Total	4859 (13.3)	-	1448 (4.1)	-	2737 (7.5)	-	539 (1.5)	-
Secondhand Smoke Exposure (Days/Week)	-	0.146	-	0.118	-	0.128	-	0.096
None	2697 (11.1)	-	631 (2.8)	-	1439 (5.9)	-	194 (0.8)	-
1–2	678 (11.3)	-	209 (3.7)	-	384 (6.4)	-	76 (1.3)	-
≥3	1484 (24.4)	-	608 (8.8)	-	914 (15.0)	-	269 (3.9)	
Family Smoking Status	-	0.062	-	0.056	-	0.052	-	0.037
No	17,511 (10.9)	-	401 (2.7)	-	953 (6.0)	-	142 (1.0)	-
Yes ^b^	3108 (15.2)	-	1047 (5.0)	-	1784 (8.7)	-	397 (1.9)	-
Father	-	0.036	-	0.026		0.028	-	0.004
No	2380 (12.2)	-	656 (3.6)	-	1332 (6.8)	-	269 (1.5)	-
Yes	2479 (14.6)	-	792 (4.6)	-	1405 (8.3)	-	270 (1.6)	-
Mother	-	0.080	-	0.105		0.068	-	0.073
No	4550 (12.8)	-	1257 (3.7)	-	2547 (7.2)	-	460 (1.3)	-
Yes	309 (29.1)	-	191 (14.7)	-	190 (17.9)	-	79 (6.1)	-
Siblings	-	0.159		0.134		0.130	-	0.097
No	4104 (12.0)	-	1119 (3.4)	-	2278 (6.6)	-	399 (1.2)	-
Yes	755 (34.8)	-	329 (14.0)	-	459 (21.2)	-	140 (6.0)	-
Grandparents	-	0.017	-	0.026	-	0.010	-	0.046
No	4498 (13.2)	-	1289 (3.9)	-	2540 (7.4)	-	445 (1.4)	-
Yes	361 (15.6)	-	159 (5.9)	-	197 (8.5)	-	94 (3.5)	-
Friends’ Smoking Status	-	0.524	-	0.499	-	0.433	-	0.383
None	208 (1.4)	-	78 (0.3)	-	123 (0.8)	-	34 (0.1)	-
Some	1614 (10.2)	-	559 (6.2)	-	705 (4.4)	-	118 (1.3)	-
Most/All	3037 (53.9)	-	811 (45.7)	-	1909 (33.9)	-	387 (21.8)	-
Witnessed Smoking at School	-	0.163	-	0.072	-	0.136	-	0.061
No	1725 (8.4)	-	685 (3.0)	-	886 (4.3)	-	228 (1.0)	-
Yes	3134 (19.6)	-	763 (6.0)	-	1851 (11.6)	-	321 (2.5)	-

Data are expressed as number (%); ^a^ All comparisons between family and friend smoking and adolescent smoking were significant (*p* < 0.05); ^b^ Any family members who smoke.

**Table 3:** Odds ratios (95% CI) for current smoking ^a^.

Characteristic	Boys		Girls	
	OR	95% CI	OR	95% CI
Secondhand Smoke Exposure in Household (/None)	-	-	-	-
1–2 Days	0.91	0.82–1.01	1.14	0.96–1.36
≥3 Days	1.90	1.75–2.07	2.06	1.80–2.35
Family Smoking Status (/No)	-	-	-	-
Yes	1.34	1.24–1.44	1.52	1.34–1.73
Father	1.17	1.09–1.25	1.11	0.98–1.25
Mother	1.93	1.64–2.28	2.62	2.15–3.21
Siblings	2.34	2.09–2.62	2.38	2.04–2.78
Grandparents	1.33	1.16–1.52	1.49	1.23–1.82
Friends’ Smoking Status (/No)	-	-	-	-
Some	5.73	4.92–6.68	11.92	9.32–15.25
Most/all	36.22	30.93–42.40	94.58	73.25–122.13
Witnessed Smoking at School (/No)	-	-	-	-
Yes	1.87	1.74–2.01	1.46	1.29–1.64

^a^ Adjusted for grade, perceived academic records, perceived socioeconomic status, alcohol drinking frequency, frequency of intense physical activity, disease history, and stress level; OR = Odds ratios; 95% CI = 95% confidence intervals.

**Table 4:** Odds ratios (95% CI) for current electronic cigarette smoking ^a^.

Characteristic	Boys		Girls	
	OR	95% CI	OR	95% CI
Secondhand Smoke Exposure in Household (/None)	-	-	-	-
1–2 Days	1.00	0.88–1.14	1.41	1.07–1.86
≥3 Days	1.96	1.78–2.17	2.86	2.34–3.49
Family Smoking Status (/No)	-	-	-	-
Yes	1.36	1.24–1.49	1.60	1.31–1.95
Father	1.16	1.07–1.26	0.92	0.77–1.09
Mother	1.84	1.52–2.22	2.46	1.87–3.24
Siblings	2.07	1.83–2.35	2.49	2.01–3.10
Grandparents	1.20	1.02–1.42	2.59	2.02–3.31
Friends’ Smoking Status (/No)	-	-	-	-
Some	3.64	2.98–4.44	6.51	4.40–9.64
Most/all	22.82	18.70–27.85	81.43	55.49–119.51
Witnessed Smoking at School (/No)	-	-	-	-
Yes	1.94	1.77–2.12	1.90	1.58–2.28

^a^ Adjusted for grade, perceived academic records, perceived socioeconomic status, alcohol drinking frequency, frequency of intense physical activity, disease history, and stress level.
